# Can Lokomat therapy with children and adolescents be improved? An adaptive clinical pilot trial comparing Guidance force, Path control, and FreeD

**DOI:** 10.1186/s12984-017-0287-1

**Published:** 2017-07-14

**Authors:** Tabea Aurich-Schuler, Fabienne Grob, Hubertus J.A. van Hedel, Rob Labruyère

**Affiliations:** 1 Rehabilitation Center Affoltern am Albis, Children’s University Hospital Zurich, Muehlebergstrasse 104, CH-8910 Affoltern am Albis, Switzerland; 2 Children’s Research Center, Children’s University Hospital Zurich, Steinwiesstrasse 75, CH-8032 Zurich, Switzerland; 3 Department of Health Sciences and Technology, ETH Zurich, Vladimir-Prelog-Weg 1-5/10, CH-8093 Zürich, Switzerland

**Keywords:** Youths, Cerebral Palsy, Neurological gait disorders, Robot-assisted gait therapy, Impedance control, FreeD motion, Surface Electromyography, Kinematic variability

## Abstract

**Background:**

Robot-assisted gait therapy is increasingly being used in pediatric neurorehabilitation to complement conventional physical therapy. The robotic device applied in this study, the Lokomat (Hocoma AG, Switzerland), uses a position control mode (*Guidance Force*), where exact positions of the knee and hip joints throughout the gait cycle are stipulated. Such a mode has two disadvantages: Movement variability is restricted, and patients tend to walk passively. Kinematic variability and active participation, however, are crucial for motor learning. Recently, two new control modes were introduced. The *Path Control* mode allows the patient to walk within a virtual tunnel surrounding the ideal movement trajectory. The *FreeD* was developed to support weight shifting through mediolaterally moveable pelvis and leg cuffs. The aims of this study were twofold: 1) To present an overview of the currently available control modes of the Lokomat. 2) To evaluate if an increase in kinematic variability as provided by the new control modes influenced leg muscle activation patterns and intensity, as well as heart rate while walking in the Lokomat.

**Methods:**

In 15 adolescents with neurological gait disorders who walked in the Lokomat, 3 conditions were compared: *Guidance Force*, *Path Control,* and *FreeD*. We analyzed surface electromyographic (sEMG) activity from 5 leg muscles of the more affected leg and heart rate. Muscle activation patterns were compared with norm curves.

**Results:**

Several muscles, as well as heart rate, demonstrated tendencies towards a higher activation during conditions with more kinematic freedom. sEMG activation patterns of the M.rectus femoris and M.vastus medialis showed the highest similarity to over-ground walking under *Path Control*, whereas walking under *FreeD* led to unphysiological muscle activation in the tested sample.

**Conclusions:**

Results indicate that especially *Path Control* seems promising for adolescent patients undergoing neurorehabilitation, as it increases proximal leg muscle activity while facilitating a physiological muscle activation. Therefore, this may be a solution to increase kinematic variability and patients’ active participation in robot-assisted gait training.

**Electronic supplementary material:**

The online version of this article (doi:10.1186/s12984-017-0287-1) contains supplementary material, which is available to authorized users.

## Background and technical introduction

Walking disorders are a common problem in patients with neurological impairments. Accordingly, robot-assisted therapy is used in neurorehabilitation to increase the dose of task-specific gait training. The most frequently applied gait orthosis is the Lokomat (Hocoma AG, Volketswil, Switzerland). The Lokomat is a robotic exoskeleton, used in rehabilitation centers to complement conventional therapies since it appears to be a feasible and promising therapeutic tool for adults as well as for children and adolescents [1–5]. Nevertheless, its effectiveness is being controversially discussed. Some studies concluded that robot-assisted therapy is superior to manual or conventional therapy in patients with stroke [6, 7] while other studies came to the opposite conclusion for stroke survivors as well as for patients with spinal cord injury [8–11]. Recent research, especially with patients following stroke, pointed out that a combination of robot-assisted therapy and conventional physical therapy might be the most promising solution [1, 3]. Thereby, robotic devices could offer a safe, simplified, and supportive environment for the therapy while also supporting visual feedback and haptic learning, which is thought to lead to the best learning performance of movements [12, 13]. However, clinical routine and scientific evidence showed that the provision of such a supportive environment comes with a price: the full and constant guidance of the robot often leads to patients being passive which might result in reduced muscle activity [14–16]. It furthermore limits active participation, dynamic walking pattern adaptation, variability in movements, and the possibility to make errors. These are all important factors for motor learning and for improving gait performance [16–20]. Patients have to train in many different ways and as often as possible (“repetition without repetition”) [21]. In an animal study, Cai et al. [22] demonstrated that spinal cord-transected mice showed a faster and a more distinct recovery when they trained with variable compared to fixed robotic trajectories for movements of the hind limbs. Although these results are encouraging, it remains unclear if these findings also translate to humans. Anyhow, improvement of rehabilitation robots, especially for functionally more advanced patients, is essential.

Consequently, new approaches have been developed that take into account the patient’s functional ability. These are based on the technical reduction of the supportive force to ensure active participation of the patient as well as to increase the possible variability of the movement [23]. The following paragraphs provide a comprehensive overview of the commercially available control soft- and hardware of the Lokomat.

### Control modes of the Lokomat

Currently, two different commercially available modes exist to quantify and modify the amount of support the patients receive during walking: *Guidance Force* and *Path Control*.

#### Guidance force

The original mode *Guidance Force* can be set from 0 to 100%. Walking at 100% guidance (impedance control) corresponds to a position-controlled mode, i.e. there is a predetermined cyclical movement trajectory for the knee and hip in the sagittal plane from which no deviation is possible [24–26]. Therefore, theoretically, no active participation of the patient is needed. From a clinical perspective, this might be a solution for severely affected patients [27].

As soon as the *Guidance Force* parameter is <100%, the impedance is reduced, which means that the restoring forces that push the patient’s hip and knee towards the reference trajectory are reduced. Therefore, small deviations from the given trajectory are allowed, and the greater the deviation is, the larger becomes the force that pushes the patient back to the trajectory (like a spring). When *Guidance Force* is set to 0%, the Lokomat will not provide support for the patient’s movements and should only compensate for robotic dynamics (gravity and Coriolis forces) but not for inertia. The downside of robotic devices using classical impedance control is the temporal restriction in walking. They hinder the patients to vary their timing without losing control in space and to experience kinematic variability in a safe and supporting way [26].

#### Path control

**Fig. 1:**
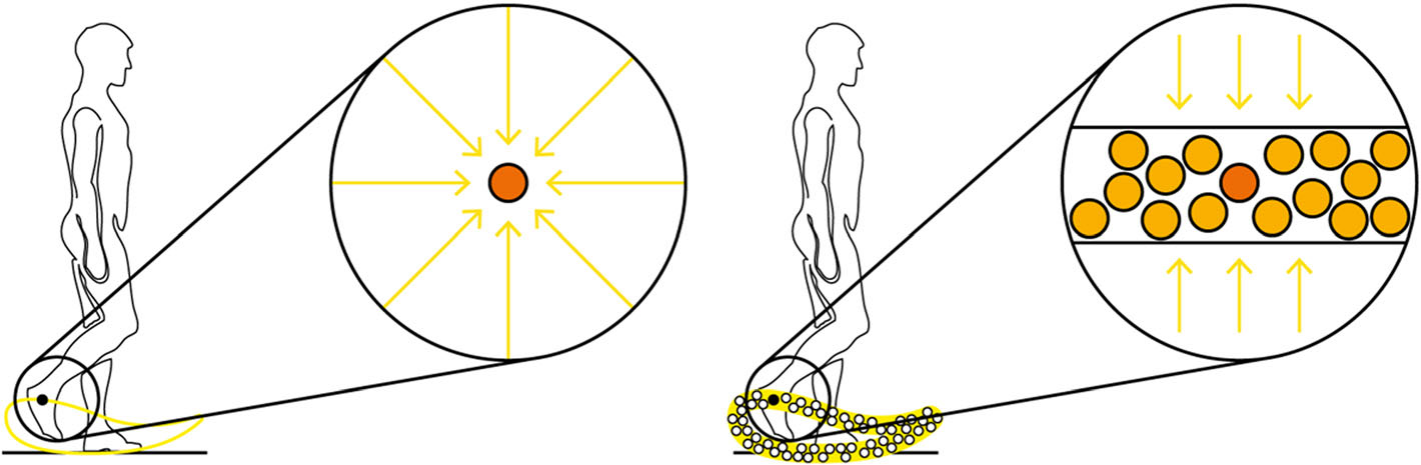
*Guidance Force* and *Path Control* mode. Left: the *Guidance Force* mode. Right: *Path Control* mode. The orange dots indicate the required position in the sagittal plane at a specific time point in the gait cycle (spatiotemporal placement). The yellow arrows represent the forces that push the patient to the reference trajectory (*Guidance Force*) or the tunnel (*Path Control*). The additional dots on the right side indicate several possible positions, symbolizing the kinematic variability. Images courtesy of Hocoma AG

The first solution to solve this issue with the limited variability in kinematics of exoskeleton robots was proposed by Cai et al. [28] in mice, and it was adapted to stroke patients by Banala et al. [29]. A virtual tunnel was implemented, in which the patients could modify their trajectories with a certain spatial and temporal freedom while a moving force supported them to conduct the movement in accordance with the treadmill speed. For the Lokomat, the first version of this strategy called *Path Control* was implemented by Duschau-Wicke et al. [26], and it has been commercially implemented in all Lokomat Pro version 6.0 devices since 2014. In *Path Control*, kinematic variability is offered by a torque field tunnel in joint space which controls the spatiotemporal characteristics of the gait pattern by applying corrective torques if the leg position is outside of the tunnel. The width of the tunnel can be set to narrow (small deviations allowed), middle, and wide (large deviations allowed). The “Support Force” (0–100%) assists the patient with the step timing. It provides an extra “wind” of force in the direction of the gait trajectory and can help the patient temporally to overcome weakness. It also reduces the uncompensated inertia of the robot (Fig. 1, for technical details, see [26, 30, 31]). Since *Guidance Force* and *Path Control* are superimposed mechanisms, the *Guidance Force* mode must be set to lower than 30% to enable *Path Control* to unfold its advantages.

Only a few studies have investigated *Path Control* in adult humans. They could show an increased active participation [30], a more physiological gait pattern [27], and improvements in clinical gait parameters when training with *Path Control* instead of *Guidance Force* [14, 32]. So far, no study exists that implemented the *Path Control* mode in children and adolescents with neurological gait disorders.

#### FreeD

Up to this stage, the pelvis and its motions were constrained to the sagittal plane and also the leg cuffs were fixed preventing lateral movements during Lokomat walking. However, lateral pelvic displacement is physiological and necessary for a natural gait pattern [33]. Different studies with robot-assisted devices concluded that restrictions in pelvic motions severely affect gait dynamics [34] and alter muscle activation patterns [8, 15] and, therefore, should be avoided.

**Fig. 2:**
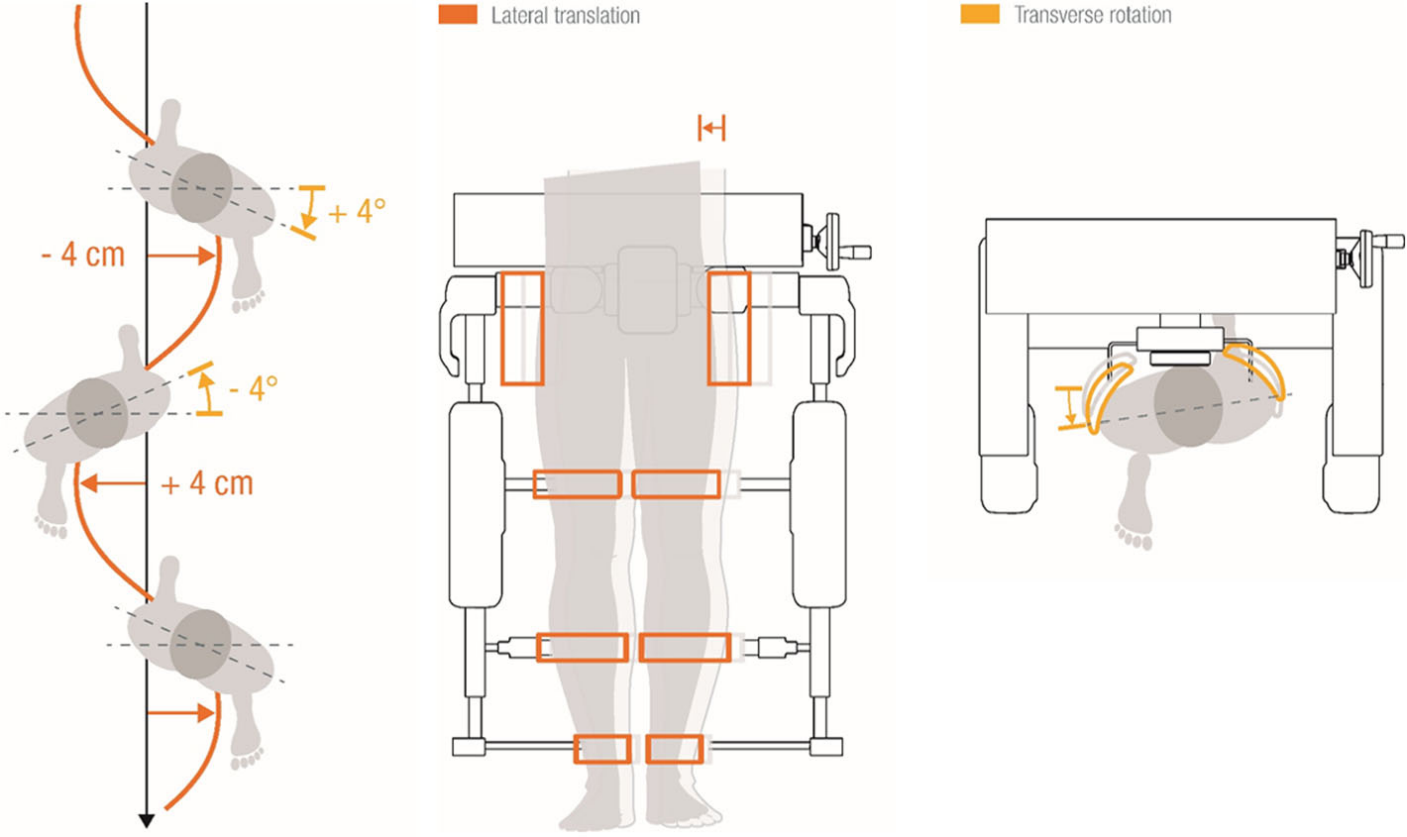
Lateral translation and transverse rotation of *FreeD.* Left: Lateral pelvis movement and rotation during physiological walking. Middle: Possibility of lateral pelvis and leg translation with the new *FreeD*. Right: Possibility of pelvis rotation with the new *FreeD*. Images with courtesy of Hocoma AG

In October 2014, a new module for the Lokomat was introduced: The *FreeD*. With this hardware and software approach, the pelvis is now movable in the frontal plane to a lateral translation of up to 4 cm (per side) and in the transversal plane to a pelvic rotation of up to 4° (per side). Additionally, the cuffs have a laterally movable range (Fig. 2). This should support the natural lateral pelvis displacement as well as weight shifting during walking and might enable an additional balance training [35] which would be useful since balance is often particularly affected in patients with neurological disorders [34]. Another study showed that with rhythmic weight-shifting training, gait performance in children with spastic diplegic cerebral palsy could be increased [36]. Therefore, the *FreeD* might be a promising renewal for robot-assisted gait training, “making the walking pattern more physiological and more natural” [35].

Although a substantial amount of work has been done during the last years in this rapidly growing field, the question concerning the most effective control algorithm for robot-assisted gait training still remains open [23]. Therefore, the aim of this study was to investigate the new control modes *Path Control* and *FreeD* in children and adolescents with neuromotor disorders and to compare them to *Guidance Force*. To examine the influence of these control modes on sEMG parameters during Lokomat walking, we formulated the following research questions: (1) Quantitative gait analysis: Does training in the Lokomat under a condition with more kinematic freedom leads to an increase in muscle activity and heart rate (*Guidance Force* < *Path Control* < *FreeD*)? and (2) Qualitative gait analysis: Does a change in muscle activity go along with maintaining a physiological gait pattern?

## Methods

### Participants

Thirty-seven in- and out-patients of the Rehabilitation Center for Children and Adolescents in Affoltern am Albis were recruited between April 2015 and 2016 to join the study. They met the following inclusion criteria: (1) a neurological impairment resulting in a gait disorder, (2) no contraindications for the training in the Lokomat (see [37]), (3) able to communicate fear, discomfort or pain, and (4) understanding simple instructions, (5) a femur length of 35-47 cm (currently, *Path Control* is only available for the adult Lokomat orthosis) and (6) written informed consent of parents and adolescents ≥14 years and assent of children <14 years. Sixteen children and adolescents agreed to participate in the study. Twenty-one disagreed due to different private reasons (e.g. distance for traveling to the clinic, busy at school). Patients were characterized by age, daily life mobility aids, and the Gross Motor Function Classification System (GMFCS level, only available for children and adolescents with cerebral palsy [38]). Measurements of the Manual Muscle Test [39] and the Selective Control Assessment for the Lower Extremity [40] were performed to determine the more affected leg of the patients. The completed STROBE checklist (see Additional file 1) and source data (see Additional files 2, 3, 4, and 5) can be found in the appendix.

### Gait training robot and control modes

Detailed information about the used Lokomat device can be found elsewhere [24, 41, 42]. In this study, the Lokomat exoskeleton was adapted to every patient individually, ensuring that walking in the robot was as comfortable as possible. Training parameters were selected by clinical experience [43]. The treadmill was set to a comfortable speed for the participant (initial speed was always 1.8 km/h and participants could then change it in small steps until they found it to be comfortable). Average speed ± standard deviation was 1.96 ± 0.15 km/h and it was kept constant during the measurements. The amount of body weight support was set to 30% of the child’s body weight. Patients had to wear elastic foot lifters to support toe clearance during swing phase and a mirror provided visual feedback of the walking pattern. To allow for a warm-up (all participants had prior Lokomat experience), each patient walked about 10 min with 100% *Guidance Force* (baseline walking setting). After this familiarization period, all patients reported that walking in the device felt comfortable and that the kinematic trajectory was easy to follow.

### Experimental design

**Fig. 3:**
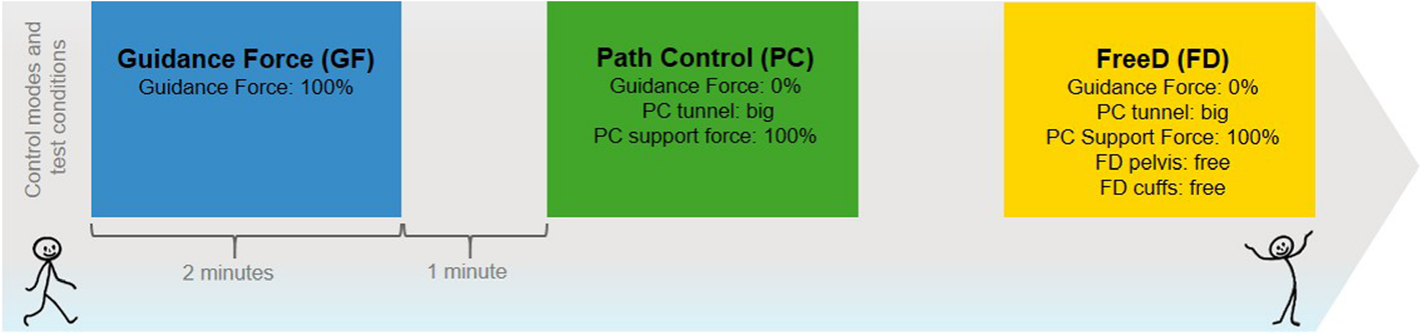
Study overview. Overview of the three different control modes and test conditions. The order was randomized and every condition lasted two minutes with a one minute break in between

The exact device settings for the three experimental conditions are depicted in Fig. 3. The order of the conditions was randomized [44], and each condition lasted 2 min. Data recording occurred during these 2 min, and standardized instructions were given before and throughout the testing (see Additional file 7). Between the conditions, a break of 1 min with the baseline walking setting allowed the patient to relax for a moment.

As already mentioned in the technical background, *Guidance Force* and *Path Control* are superimposed control modes, which optionally can be combined with the *FreeD* module. Therefore, condition GF was a standard therapy setting of 100% *Guidance Force (see blue box in* Fig. 3 *)*. Condition PC was a setting with higher kinematic freedom (*Path Control*), whereby *Guidance Force* was set to 0% to allow *Path Control* to completely take over *(see green box in* Fig. 3 *)*. To max out the Lokomat’s kinematic freedom, condition FD was a combination of *Path Control* and *FreeD*, whereby the pelvis and cuffs were moveable (pelvis free up to 4° of rotation and 2 cm lateral shift to each side; cuffs free, see yellow box in Fig. 3). In both, *Path Control* and *FreeD*, the Support Force within *Path Control* was set to 100% (see green and yellow boxes in Fig. 3).

The study consisted of further sub-conditions where the Support Force during *Path Control* was modulated (comparable to [31]), and the degrees of freedom (pelvis and cuffs) of the *FreeD* were modified, but to remain concise, they are not part of this manuscript.

### Measurements

**Fig. 4:**
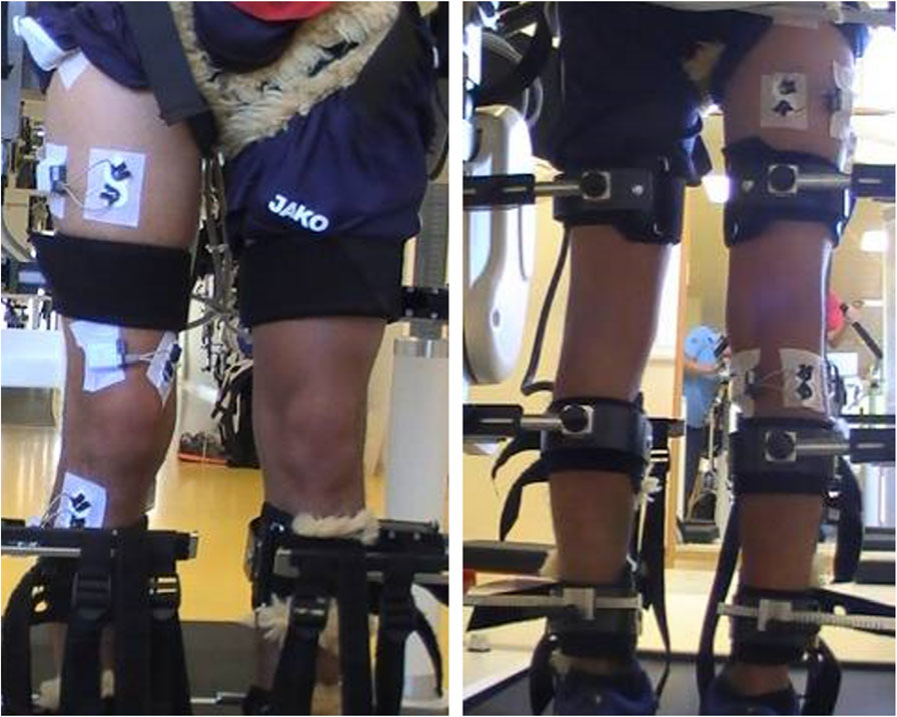
sEMG electrodes placement. The placement of the electrodes (according to the SENIAM guidelines, [46]). Left (from top to bottom): M.rectus femoris, M.vastus medialis, M.tibialis anterior. Right (from top to bottom): M.biceps femoris long head, M.gastrocnemius lateralis

The measurements took place at the Rehabilitation Center for Children and Adolescents in Affoltern am Albis, Switzerland. The experimental protocol was in accordance with the Declaration of Helsinki [45] and was approved by the Ethical Committee of the Canton Zurich, Switzerland. Activity of the following 5 muscles of the more affected leg was determined by surface electromyography (sEMG): the M.rectus femoris (RF), M.vastus medialis (VM), M.biceps femoris, long head (BF), M.tibialis anterior (TA) and M.gastrocnemius lateralis (GL). The surface electromyography recordings were done with the Wireless TeleMyo DTS system and the MyoResearch XP software (Noraxon Inc., Scottsdale, USA). The system was time-synchronized with a video camera that was positioned beside the child’s measured leg to identify gait cycle events. The placement of the sEMG electrodes was always done by the same therapist for all measurements adhering as closely as possible to the SENIAM guidelines [46] (Fig. 4). The skin was prepared by shaving and applying an abrasive paste and then self-adhesive Ag/AgCl snap electrodes (Noraxon Dual Electrodes, 10 mm diameter and 20 mm inter-electrode distance, Noraxon Inc., Scottsdale, USA) were positioned. The quality of the sEMG signals was visually inspected during the familiarization period as well as during the measurements to ensure that the electrodes were correctly placed and to exclude movement artifacts during walking.

Furthermore, a heart rate belt (Polar RS800CX Pro Training, sampling frequency 0.2 Hz, Polar Electro Oy, Kempele, Finland) recorded the heart rate during training. As the *Guidance Force* condition acted as theoretical baseline of physiological walking, we wanted to know if participants could maintain this physiological pattern when switching to other control strategies. Therefore, after each walking condition (see Fig**.** 3), the therapist scored the walking pattern as “physiological” (=1) or “not physiological” (=0). This decision was subjective, but it was mainly based on the fulfillment of the following factors: timing of heel strike, knee extension in stance phase, step symmetry, step length, toe clearance, and rhythm. If two or more factors were conspicuous, the performance was rated as “not physiological”. Additionally, the patient scored the experience as “comfortable” (=1) or “not comfortable” (=0), as child-friendly terms for “physiological” and “not physiological”. This was differentiated from signs or statements of physical discomfort or even pain, which led to a temporary halt of the experiment.

### Data analysis

sEMG data were rectified and smoothed by a Root Mean Square with a time window of 100 ms. For the sEMG-data analysis, 10 strides [47] after 30s of the start of each condition were analyzed. The markers for heel strike and toe off were set automatically by the program. Every single marker was controlled and checked visually with the synchronized video recording and adjusted manually if necessary. Afterward, the sEMG data were exported to Matlab (Matlab 7.1, the MathWorks Inc., Natick MA, USA). For the analysis investigating quantitative changes in sEMG activity, the sEMG of the 10 stance- and swing phases of each muscle for every condition of each patient were averaged. Then, they were merged and time-normalized to a 100% gait cycle (1000 samples).

For the analysis investigating qualitative changes in sEMG activity, we took the sEMG individual time-normalized averaged profile of each muscle for every condition. These gait curves were then amplitude-normalized to its maximal value (max. value = 100%). Afterwards, we took the mean gait cycle over all participants per condition to generate grand-averaged gait cycle profiles for each muscle per condition. Based on these grand averages, we determined “On-”and “Off-phases” for each muscle. A muscle was considered to be ‘on’ if its activity exceeded a threshold, which was set at the minimum amplitude of the sEMG grand average per muscle plus two standard deviations (SD) (adapted from [48]). Then, these on- and off-phases were compared to the on- and off-phases of sEMG norm curve data from 87 typically developing children from Chang et al. [48], digitized with the WebPlotDigitizer (retrieved from [49]). Comparisons were made with two metrics: (i) the Spearman correlation coefficient that indicates the similarity with the norm curve; and (ii) the percentage of overlap as an indicator for the “normality” of the muscle activation pattern. It was calculated as: $\frac{correct\  ON\  overlap\ \left[\%\right]+ correct\  OFF\  overlap}{correct\  ON\  overlap\ \left[\%\right]+ false\  ON\  overlap\ \left[\%\right]+ correct\  OFF\  overlap\ \left[\%\right]+ false\  OFF\  overlap\ \left[\%\right]}$

For the heart rate, the values for the 2 min were averaged for each condition.

### Statistics

The statistics were done with IBM SPSS Statistics 22 (IBM Corporation, Armonk, NY, USA). Data were checked for normal distribution with the Shapiro-Wilk test together with Q-Q-plots and histograms. Because most of the data were not normally distributed, subsequent qualitative and quantitative analyses were done with non-parametric tests (Spearman correlations, Friedman- and Wilcoxon-tests). The significance level was set at α = 5%. Post-hoc corrections for multiple testing were done by applying False Discovery Rate corrected *p*-values (FDR, [50]). Additionally, effect sizes were calculated and scored according to Cohen’s benchmarks (d = 0.2 is small, d = 0.5 is medium, and d = 0.8 is considered a large effect size, [51]). The correlations of the sEMG comparisons were interpreted as follows (adopted from Evans, [52]): *r* < 0.20, “very weak”; 0.20–0.39, “weak”; 0.40–0.59, “moderate”; 0.60–0.79, “strong” and 0.80–1.00 “very strong relationship”.

## Results

Fifteen patients (5 girls, 10 boys) with a mean age of 16 ± 2y completed the trial. Details about the patients’ characteristics are listed in Table 1. In one participant, the measurements had to be stopped immediately after the beginning for safety reasons (patient ID 13), as the therapist noted during the robot-walking familiarization period that the patient would not be able to walk with less than 100% *Guidance Force*.

**Table 1:** Patients' characteristics

ID	Age (years)	Main diagnosis (GMFCS Level)	More impaired leg	Walking speed (km/h)	Daily life mobility aids
1	19	CP, bilateral ataxic (III)	right	2.2	Dorsal walking frame for longer distances
2	19	Hereditary spastic paraplegia	right	2.2	None
3	14	CP, bilateral ataxic (II)	left	2.0	Dorsal walking frame, ankle-foot orthoses
4	14	ABI ^a^ (unilateral paresis)	left	1.9	Foot-up orthosis
5	16	ABI ^a^ (unilateral spastic paresis)	left	2.0	None
6	19	CP, bilateral spastic (III)	right	2.0	Crutches, orthopedic shoes
7	13	CP, bilateral spastic (II)	left	2.0	Foot-up orthosis
8	15	MMC L3/L4	right	1.8	Crutches
9	16	CP, bilateral spastic (II)	right	1.8	Ankle-foot orthoses
10	16	CP, bilateral spastic (III)	left	1.9	Dorsal walking frame for longer distances, ankle-foot orthoses
11	14	CP, bilateral spastic (III)	right	2.1	Ankle-foot orthoses
12	20	ABI ^a^ (bilateral spastic paresis)	right	1.8	None
13	19	CP ^c^, bilateral spastic (IV)	right	1.4	Wheelchair, ankle-foot orthoses
DROP-OUT					
14	15	CP, unilateral spastic (I)	left	1.8	None
15	14	ABI ^a^ (unilateral spastic paresis)	left	1.8	None
16	12	ABI ^b^ (unilateral paresis)	left	2.1	None

*Abbreviations*: *CP* cerebral palsy, *GMFCS* Gross Motor Function Classification System [38], *MMC* Meningomyelocele (spina bifida), *ABI* acquired brain injury, ^a^= measurements more than 2.5 years after event, ^b^= measurements 2 months after event. ^c^ID 13 was excluded because the measurements had to be stopped shortly after the beginning

Since some of the participants had to wear lower leg orthoses for ankle-stabilization, we had missing data of the M.tibialis anterior for 2 participants and the M.gastrocnemius lateralis for 3 participants, respectively.

### Quantitative changes in sEMG activity and heart rate

**Fig. 5:**
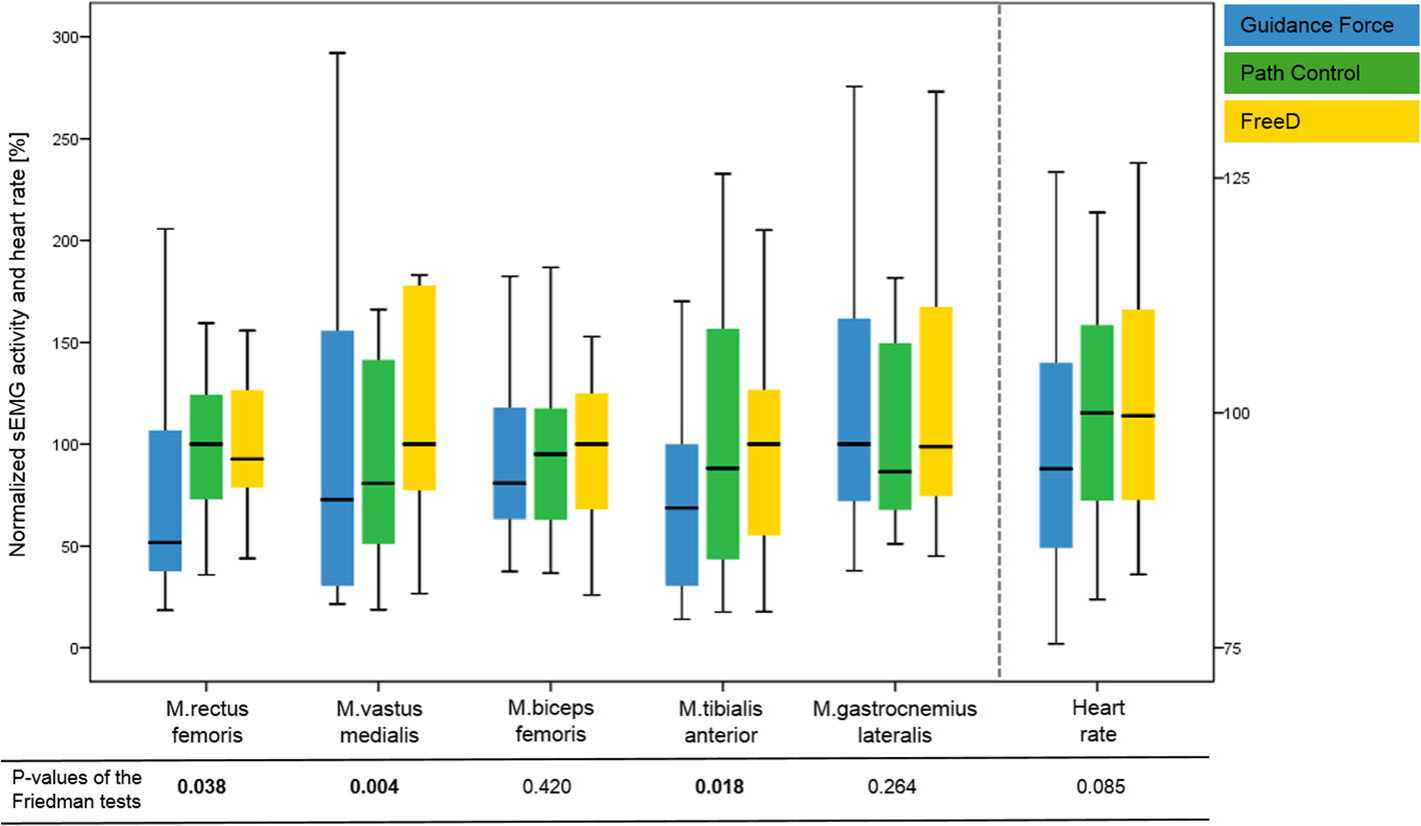
sEMG amplitudes and heart rate during walking under the three different control modes. To facilitate a comparison, the conditions were normalized by setting the highest median sEMG activity value of the three walking conditions for each muscle to 100% (and the same for heart rate). To improve visualization, outliers are not shown in the figure. However, they are included in the statistical analyses. *P*-values of the Friedman tests for each muscle and for heart rate are shown below the graph. Statistically significant data are indicated in bold

We found an increase in quantitative muscle activation in several muscles when the kinematic freedom of the Lokomat was enlarged. Significant group differences between the three conditions could be found for the M.rectus femoris (*P* = 0.038), the M.vastus medialis (*P* = 0.004) and the M.tibialis anterior (*P* = 0.018). Heart rate did not reach significant results but a trend could be detected (*P* = 0.085). sEMG amplitudes, the heart rate, and the *p*-values of the 3 conditions and the five muscles are presented in Fig. 5.

Additional file 7: Fig. S1 shows that the chosen protocol was adequately timed to approximately allow heart rate to reach a steady state during the conditions and return to baseline during the breaks.

Significant differences in quantitative muscle activation were found for M.rectus femoris, M.vastus medialis and M.tibialis anterior. Pairwise post-hoc comparisons with effect sizes of the different conditions are presented in Table 2.

**Table 2:** Comparison of the sEMG amplitudes between conditions. *P*-values of the Wilcoxon tests before and after FDR correction for multiple testing and effect sizes are shown

	Comparisons	*p*-values of the Wilcoxon test	FDR corrected *p*-values	Effect sizes
M.rectus femoris	GF - PC	0.100	0.150	−0.30
	GF - FreeD	0.061	0.150	−0.34
	PC - FreeD	0.496	0.496	−0.13
M.vastus medialis	GF - PC	0.173	0.173	−0.25
	GF - FreeD	**0.020**	**0.030**	−0.43
	PC - FreeD	**0.002**	**0.006**	−0.56
M.biceps femoris	GF - PC	0.955	0.955	−0.01
	GF - FreeD	0.363	0.545	−0.17
	PC - FreeD	0.125	0.375	−0.28
M.tibialis anterior	GF - PC	0.133	0.199	−0.30
	GF - FreeD	**0.023**	0.069	−0.45
	PC - FreeD	0.701	0.701	−0.08
M.gastrocnemius lateralis	GF - PC	0.209	0.582	−0.26
	GF - FreeD	1.000	1.000	0.00
	PC - FreeD	0.388	0.582	−0.18
Heart rate	GF - PC	0.069	0.104	−0.33
	GF - FreeD	**0.047**	0.104	−0.36
	PC - FreeD	0.427	0.427	−0.15

*Abbreviations*: *FDR* False Discovery Rate [50], *GF* Guidance Force, *PC* Path Control. Statistically significant data are indicated in bold

### Qualitative changes in sEMG activity and walking patterns

**Fig. 6:**
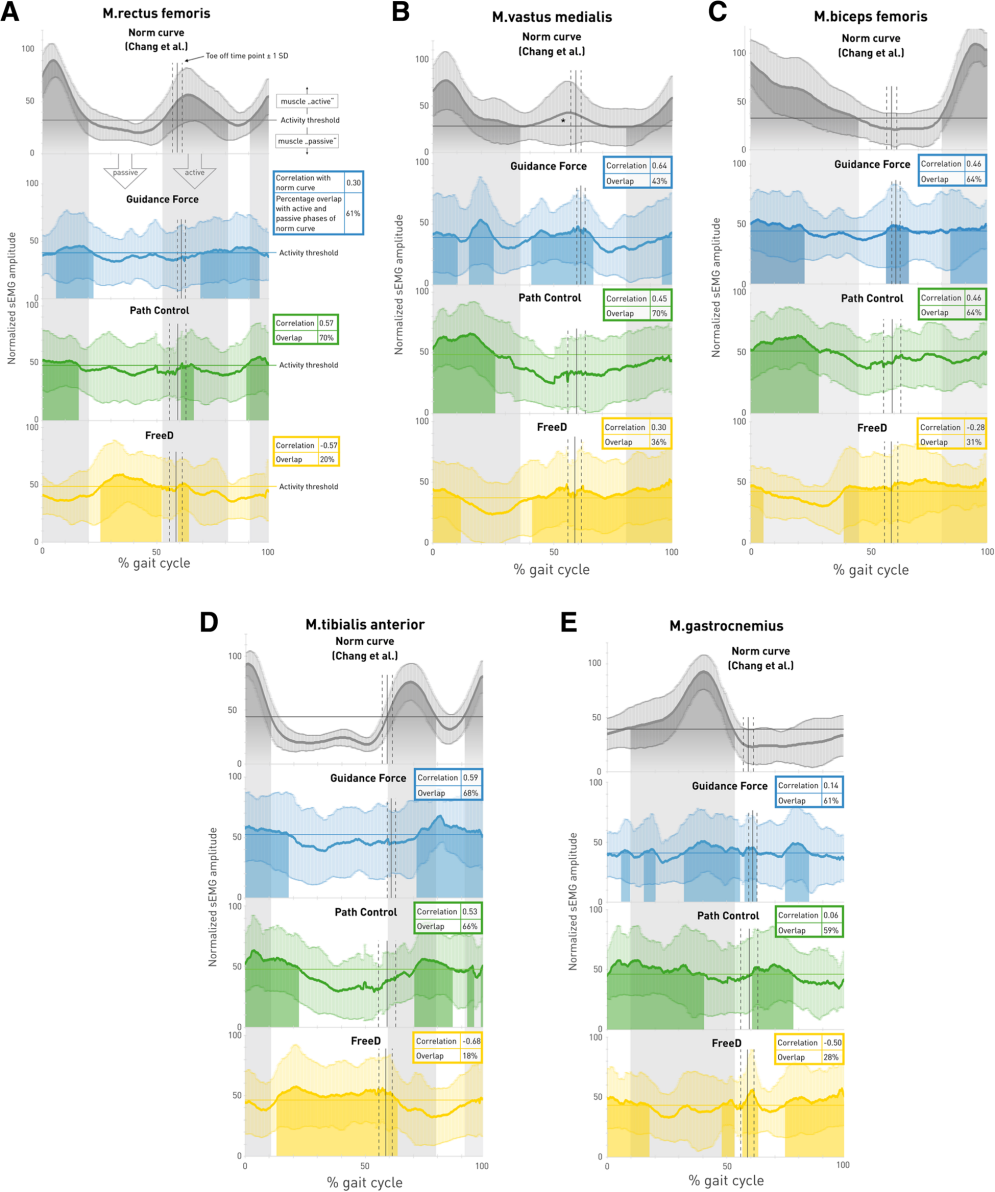
Grand-averaged gait cycle sEMG profiles for each muscle and each condition. Linear envelope curves of the averaged gait cycle per muscle show mean ± standard deviation of the norm curve (adapted from Chang et al. [48]) and the three different walking conditions: *Guidance Force* (blue), *Path Control* (green) and *FreeD* (yellow). Muscle onset threshold was defined as 2 standard deviations above the minimum amplitude of the mean curve over all patients for each muscle separately. Grey banners in the background indicate that the muscle is expected to be “active” (= norm curve activity above the threshold); white banners in the background indicate that the muscle is expected to be “passive” (= norm curve activity below the threshold), see Fig. 6a. Colored shadows indicate that the muscle during that timepoint in the specific walking condition is active. Toe off time and shift from stance to swing phase is indicated with a vertical line ± one standard deviation (dashed lines). The “correlation” value refers to the Spearman correlation of the pattern of that specific walking condition with that of the norm pattern and “overlap” indicates the percentage of “activity” and “passivity” overlap of the pattern of that specific walking condition with that of the norm curve. *According to Chang et al. [48], the M.vastus medialis is not active here, despite supra-threshold activity

The grand-averaged gait cycle profiles for each muscle and each condition are displayed in Fig. 6 together with reference curves of normally developing children adapted from Chang et al. [48].

Focussing on the therapist’s and patient’s scorings, the following results could be observed: During the *Guidance Force* condition, the therapist scored the gait pattern as “physiological” for all 15 patients while 13 patients rated the condition to be “comfortable” (15/13). These numbers were 8/9 during *Path Control *and 7/10 during *FreeD motion*, respectively. Apparently, all of the patients walked physiologically during the *Guidance Force* condition, whereas fewer patients were scored with a physiological gait pattern during *Path Control* and *FreeD* conditions. Table 3 summarizes the correlations and overlaps of the sEMG patterns with the norm curves and presents the therapist’s and patients’ scorings.

**Table 3:**
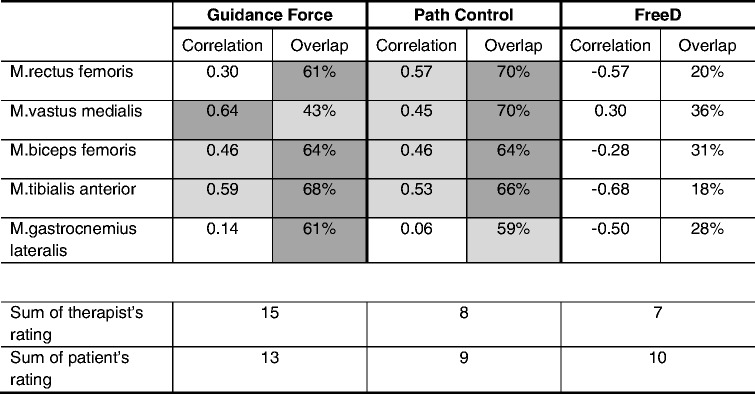
Overview of the sEMG correlations and overlaps with the norm curves and subjective ratings of the therapist and patients

Therapists had to score the patient’s walking under a specific condition as “physiological” (=1) or “not physiological” (=0); Patients had to score the walking under a specific condition as “comfortable” (=1) or “not comfortable” (=0). The color codes are adapted in accordance to the interpretation for the correlations [52]: “very weak” (or negative) and “weak” = white, “moderate” = light grey and “strong” = dark grey

## Discussion

### Quantitative changes in sEMG activity and heart rate

Our study investigated changes in muscle activity levels and patterns induced by two new hard−/software approaches which were developed to increase patients’ kinematic variability. In general, we could show partially significant increases in muscle activity and heart rate from the condition *Guidance Force* to *Path Control* (except for M.gastrocnemius lateralis) and from *Path Control* to *FreeD motion* (except for M.rectus femoris and heart rate) (Fig. 5 and Table 2). This is in line with earlier results from Duschau-Wicke et al. [30] and Schück et al. [27], where patients walked more actively under the *Path Control* mode. However, in contrast to the results from Schück et al. [27], our results could detect only partially significant trends. This distinction could be explained by the different patient population or by the fact that their controls walked on a treadmill whereas we compared our data with norm curves of free overground walking. Another reason might be that we evaluated only one training session whereas they analyzed the change over 16 training sessions. Furthermore, van Kammen et al. [53] concluded that a higher guidance force in the Lokomat in general reduced the amplitude of muscle activity (M.biceps femoris, M.gastrocnemius medialis), and that this effect depended on body weight support and gait speed. However, they observed this effect only in muscles related to stability and propulsion and not leg loading (M.vastus lateralis) and foot clearance (M.tibialis anterior). In general, only small effect sizes could be found in the analysis of quantitative changes in sEMG and heart rate, except for the M.vastus medialis, where the significant differences in muscle activation were supported by a moderate effect size (i.e. -0.56, Table 2). It should be mentioned that the economics of a neurologically impaired gait (especially in cerebral palsy) are often affected by a poor biomechanical alignment. This means that a patient with cerebral palsy commonly walks with a high sEMG activity and heart rate. Therefore, a higher sEMG activity should not be a general goal of a therapy. Rather, it must be interpreted in combination with a pattern analysis.

### Qualitative changes in sEMG activity and walking patterns

A deeper insight in the sEMG grand-averaged gait cycle profiles (Fig. 6) revealed that the interpretation of the results is not as simple as it seems. The normal activity of M.rectus femoris (Fig. 6a) has two active phases, one at the end of preswing till midswing (55–80% Gait Cycle (GC) [48]) and the other from terminal swing till the end of loading response (90–15% GC) with a clear peak in loading response. In our study, walking under *Path Control* revealed the best overlap (70%) with these active and passive phases as well as a moderate correlation (*r* = 0.57) with the pattern. This finding stands in contrast to Duschau-Wicke et al. [54] where M.rectus femoris presented an unphysiological gait pattern during walking with *Path Control*.

The M.vastus medialis normally activates in terminal swing till early midstance (86–20% GC, Fig. 6b). At first sight, the pattern during walking under *Guidance Force* in our results looks the most similar to the norm curve, but it shows an abnormal active peak in late midstance. This is in contrast to the percentage overlap, which was best for the *Path Control* condition, i.e. 70%. This high number has originated from the overlap of an active phase in initial contact and loading response (early stance phase) and a passive phase from midstance to the end of midswing. It might be confusing that the norm curve maps an additional preswing and initial swing activity (45–65% GC, * in Fig. 6b), which is being ignored in the physiological activity range determined by Chang et al. [48]. This additional activity (in this study during walking under *Guidance Force* and *FreeD*) might stem from a co-contraction for the stabilization of the knee before entering the swing phase and was also reported in previous studies [55, 56]. If this additional peak had been included by Chang et al. [48], walking under *Guidance Force* would have gained the best agreement between the two patterns (which is already visible with the current strong correlation *r* = 0.64).

The active range of M.biceps femoris is from the end of midswing till terminal stance (80–50% GC, Fig. 6c). In our study, this was best represented by both *Guidance Force* and *Path Control* with a moderate correlation (*r* = 0.46) and an overlap of 64%.

M.tibialis anterior is characterized by two peaks of activity, one from preswing till midswing (55–80% GC) and a second in terminal swing until the end of loading response (90–15% GC, Fig. 6d). In this muscle, both walking under *Guidance Force* as well as under *Path Control* could reach an acceptable overlap (68% and 66%, respectively), with moderate correlations. An abnormally low sEMG activity in terminal swing as detected in our data was also reported in other studies with children [56, 57] and could stem from the use of foot lifters [15].

The M.gastrocnemius lateralis normally presents activity from the end of the loading response to the middle of preswing (10–55% GC), with a clear peak at 40% GC (Fig. 6e). Our data could not detect this clear pattern with only very weak correlations during *Guidance Force* (*r* = 0.14) and *Path Control* (*r* = 0.06). Main functions of this muscle are to extend and stabilize the knee during stance and to induce the push off. When walking in the Lokomat, the cuffs and the robot itself take over these parts. A pattern without clear activity peaks of the M.gastrocnemius is also known from treadmill walking, where the push off is not as clear due to the moving treadmill [56]. An early onset of swing and a prolonged activity in stance are known as the plantar flexion-knee extension couple to control the second rocker and an upright position [56]. In our data, this was observable in *Path Control* and *FreeD* conditions.

In contrast to the norm data, our pattern curves lack clearly discernible peaks. At this point, it must be mentioned that Chang et al. [48] generated their norm curves by including only EMG patterns which showed the typical one or two peaks in the expected period (positive selection bias). It is obvious that the elimination of all other patterns resulted in curves with clear peaks and clear active and passive phases. In contrast, our grand-average profiles included all data. Therefore, it is difficult to get good correlations with these norm patterns, and we decided to add the overlap analysis. Thereby, it is possible to observe windows of abnormal activity, similarly to the work of van Kammen et al. [53]. This abnormal activity may indicate efforts e.g. to overcome constraints from the robot [15, 16] or increased balance needs [53, 58].

In summary, proximal leg muscle activity patterns did have the best overlaps during walking under *Path Control*, with also good patterns under *Guidance Force*. Distal leg muscles showed conflicting outcomes. While M.tibialis anterior generated quite good patterns concerning correlation and overlap, weaker correlations were found for M.gastrocnemius lateralis. Generally, during the *FreeD* motion condition, all pattern correlations were weak and mostly negative. However, *FreeD* was the only condition, where significantly higher muscle activity was found in the M.vastus medialis compared to *Guidance Force* or *Path Control*. The higher muscle activation could be a result of the efforts of the patients, who tried to compensate (unphysiologically) for too much kinematic freedom. Due to this kinematic freedom, we initially assumed that FreeD could facilitate physiological walking since patients would not be restricted in their gait trajectory. Hidler and Wall [15] indicated in their study that muscle activity patterns are altered while walking under full guidance because patients work against the robot (M.rectus femoris, M.biceps femoris). According to Ayoagi et al. [59], it is important for a natural human gait that robot-assisted devices allow the leg to swing out to the parasagittal plane and to allow the pelvis to rotate and to make a lateral translation. However, it seems that patients were unable to deal with the high kinematic freedom and therefore performed with too much muscular activity in an unnatural activation pattern to keep up the biomechanical alignment. It seems that this possibility to move to the parasagittal plane was the biggest challenge for our patients since the Guidance Force and Support Force vectors only operate in the sagittal plane.

Consequently, not only the sEMG analysis revealed issues with the kinematic freedom. Based on the therapist’s rating we saw that not all of our patients could deal with the additional kinematic freedom during *Path Control* and *FreeD* and around half of the patients rated walking in these conditions as “uncomfortable”. They developed an unphysiological gait pattern and would have needed more guidance from the Lokomat, or from the therapist through instructions. Due to the standardized instructions during the study (see Additional file 6), we refrained from this permanent verbal input. Nevertheless, these results were unanticipated since patients still received 100% Support Force while walking with *Path Control* and *FreeD*. Moreover, patients reported very differently about their impression of the kinematic variability. While some of them found the freedom comfortable, others felt unguided and actively tried to deal with this variability. It seems clear that these feelings depend on the skills of the individual patient and these results are in line with the findings of Duschau-Wicke et al. [30]. Therefore, a further analysis of varying Support Forces may give additional information about the effects on muscle activity, activation patterns, and the rating of therapists and patients.

### Clinical implications

For clinical practice in neurorehabilitation, it is important to train restorative rather than compensatory patterns [56, 60]. Therefore, inducing physiological gait patterns by training under *Guidance Force* or *Path Control* might be the preferred method. Nevertheless, our results reinforce the opinion that the therapist plays a crucial role during robot-assisted gait training during rehabilitation [43, 56]. The therapist has to decide individually which control mode is reasonable for a training with a specific patient. Certainly, this decision depends also on the aim of the training. If the goal is to train a specific, symmetric gait pattern (often the target in an early phase of rehabilitation or in severely impaired patients) then the training should be performed using the *Guidance Force* mode where the patient gets an exact and symmetric, predetermined gait trajectory. If the goal is for example to train muscle strength, endurance, joint control, weight shifting, balance, or step variability, then *Path Control* or even *FreeD* might be a good choice. The therapist’s decision to choose one or the other training option also depends on the duration with which a specific control mode is applied. A control mode with high kinematic freedom might be selected to work on a specific impairment, however, it will be difficult for the patient to train with a physiological walking pattern for a prolonged duration. In this case, the patient should get frequent, intensive verbal instructions.

### Limitations

Our patient group was a convenience sample and heterogeneous regarding diagnosis, age, level of cognition, and skills. This may have increased the data variability and influenced the results, but the aim of this study was to reflect clinical everyday life with neurological patients where the population is very heterogeneous. Since the sample size in this study is small, we should be cautious in generalizing the results of this study to similar population groups, even though the uniformity of Lokomat therapy and the standardized measurement procedure provide a highly reproducible setting. Accordingly, further studies are needed to investigate disease-specific patterns into further detail to get a finegrained impression about the effects of the different control modes on the muscle activation patterns of patients with specific neuromotor disorders.

We compared sEMG-patterns of patients with neuromotor disorders to those of a published sample of healthy controls. While an intra-individual comparison would be desirable, these patients are not able to generate a physiological gait pattern overground. Therefore, our EMG data might partially differ from the EMG norm data, e.g. regarding exact electrode placement or data preprocessing. Additionally, we cannot exclude that the 10 min-long warm-up phase with 100% *Guidance Force* influenced the performance during the test conditions.

In our study, only adolescents could participate, as *Path Control* and *FreeD* are only available for the adult Lokomat exoskeleton. As soon as a pediatric version is available, the study should be extended to children as well. Furthermore, for safety reasons, the patients had to wear foot lifters while walking in the Lokomat and it is possible that this has influenced muscular activity, especially of the distal muscles.

Another limitation is that the treadmill speed in our patients varied between 1.8–2.2 km/h to allow for comfortable walking. Since gait speed has an influence on the gait pattern [61], treadmill speed was kept constant throughout the whole experiment. Therefore, future strategies to increase patients’ interactivity with robot devices should combine the tested modes of this study with approaches to adapt the treadmill speed according to the patient’s intention [26, 62]. The results of *FreeD* in this study were against our expectations, and further research is necessary to clarify this topic. We assume that the chosen settings of the control modes might have overly affected the difficulties of the walking conditions (e.g. *Guidance Force* set to 0% and Support Force to 100% in *Path Control* and *FreeD*, see Fig. 3). This is supported by the fact that *Guidance Force* had to be adjusted to 10% in 3 patients to enable 2 min of constant walking. Nevertheless, the idea of the experiment was to select 3 conditions with differing kinematic variability. Further studies should test the *FreeD* in combination with a bigger underlying *Guidance Force* (e.g. above 60%), which will most likely be used in a clinical setting. Additionally, future studies should investigate changes of gait pattern over time (e.g. over 15 min. Walking time with the same conditions) and alterations/adaptations of the gait pattern after several training sessions with new technologies (including studies of effectiveness).

## Conclusion

With this study, we could show that alterations in muscle activity (amplitude and pattern) can occur when different control modes are used during training with the Lokomat. Therefore, it seems that patient-cooperative tools are able to address the main point of criticism against robot-assisted gait training: the passivity of the patient. Additionally, especially with *Path Control*, patients can train walking in an active and physiological way. Further studies should clarify, why the *FreeD* as tested in this study seems to be less applicable for physiologic walking in moderately affected children and adolescents with neurological gait disorders and which requirements patients must meet to train physiologically also with *FreeD*. Furthermore, longitudinal studies should be performed to address the effectiveness and long-term effects of these new control modes. However, the therapist is still the most important factor for evaluating and influencing the performance during robot-assisted gait therapy. It is his/her responsibility to guide the training process and to choose an adequate training mode to reach patients’ individual aims and goals of gait rehabilitation.

## Additional files

Additional file 1: STROBE Statement checklist. (DOCX 20 kb)

Additional file 2: Source data sEMG Guidance Force. (XLSX 27966 kb)

Additional file 3: Source data sEMG Path Control. (XLSX 27288 kb)

Additional file 4: Standardized instructions. (DOCX 14 kb) Source data sEMG FreeD. (XLSX 27602 kb)

Additional file 5: Source Data heart rate. (XLSX 107 kb)

Additional file 6: Standardized instructions. (DOCX 14 kb)

Additional file 7: Figure S1. Time course of heart rate during conditions. Time course of the average normalized heart rate curves of the 3 conditions. Thereby, the individual mean heart rate over the last minute of the warm-up phase (regular walking with 100% Guidance Force) served as an individual baseline (=100%) to which the longitudinal curves were normalized. The analysis shows that a steady state was reached after approximately 1 min and that the 1-min break was long enough for the heart rate to return close to baseline. (TIFF 1906 kb)

## Abbreviations

BF: M.biceps femoris
FDR: False Discovery Rate
GL: M.gastrocnemius lateralis
GMFCS: Gross Motor Function Classification System
RF: M.rectus femoris
SD: Standard deviation
sEMG: Surface electromyography
TA: M.tibialis anterior
VM: M.vastus medialis
