# Supplementary material for: Can Lokomat therapy with children and adolescents be improved? An adaptive clinical pilot trial comparing Guidance force, Path control, and FreeD
Source: J Neuroeng Rehabil. 2017 Jul 14;14:76. doi: 10.1186/s12984-017-0287-1 (PMC5513325; doi:10.1186/s12984-017-0287-1)
Supplement: Supplementary file 6 — Standardized instructions. (DOCX 14 kb) [file 12984_2017_287_MOESM6_ESM.docx]

# Standardized instructions

In general, the patients were instructed to match the predefined gait pattern of the Lokomat and to walk as naturally as possible throughout all conditions. Additional standardized instructions were given only if necessary (if the therapist decided that a walking pattern was no longer physiological).

Standardized instructions before a condition started:

- “please don’t worry when changes in technical support occur”
- “just try to walk as naturally as possible for the next 2 minutes”

Possible, standardized instructions after 30 seconds into the measurement:

- “try to actively complete the swing phase”
- “try to extend your knees at the end of the swing phase”
- “try to control the heel strike and put the heel softly onto the treadmill”
- “extend your knees during stance phase”
- “great, go on” (always given)

Possible, standardized instructions after 60 seconds into the measurement:

- “try to actively complete the swing phase”
- “try to extend your knees at the end of the swing phase”
- “try to control the heel strike and put the heel softly onto the treadmill”
- “extend your knees during stance phase”
- “great, go on. You’re already halfway through” (always given)

Standardized instructions at the end of a condition:

- “great job, now you have a short break, and you can let the Lokomat walk”
