# Supplementary figures and images for: Can Lokomat therapy with children and adolescents be improved? An adaptive clinical pilot trial comparing Guidance force, Path control, and FreeD
Source: J Neuroeng Rehabil. 2017 Jul 14;14:76. doi: 10.1186/s12984-017-0287-1 (PMC5513325; doi:10.1186/s12984-017-0287-1)

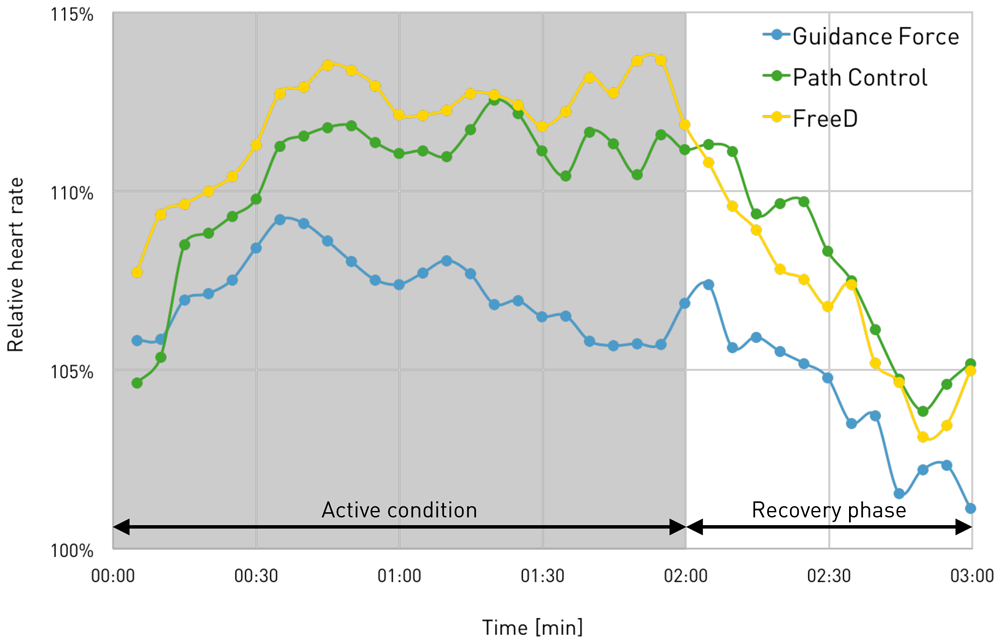

Supplement: Supplementary file 7 — Time course of heart rate during conditions. Time course of the average normalized heart rate curves of the 3 conditions. Thereby, the individual mean heart rate over the last minute of the warm-up phase (regular walking with 100% Guidance Force) served as an individual baseline (=100%) to which the longitudinal curves were normalized. The analysis shows that a steady state was reached after approximately 1 min and that the 1-min break was long enough for the heart rate to return close to baseline. (TIFF 1906 kb) [file 12984_2017_287_MOESM7_ESM.tif]
